# Listeriolysin S, a Novel Peptide Haemolysin Associated with a Subset of Lineage I *Listeria monocytogenes*

**DOI:** 10.1371/journal.ppat.1000144

**Published:** 2008-09-12

**Authors:** Paul D. Cotter, Lorraine A. Draper, Elaine M. Lawton, Karen M. Daly, David S. Groeger, Pat G. Casey, R. Paul Ross, Colin Hill

**Affiliations:** 1 Department of Microbiology, University College Cork, Cork, Ireland; 2 Alimentary Health Ltd., Cork, Ireland; 3 Alimentary Pharmabiotic Centre, University College Cork, Cork, Ireland; 4 Moorepark Food Research Centre, Teagasc, Moorepark, Fermoy, Cork, Ireland; Institut Pasteur, France

## Abstract

Streptolysin S (SLS) is a bacteriocin-like haemolytic and cytotoxic virulence factor that plays a key role in the virulence of Group A *Streptococcus* (GAS), the causative agent of pharyngitis, impetigo, necrotizing fasciitis and streptococcal toxic shock syndrome. Although it has long been thought that SLS and related peptides are produced by GAS and related streptococci only, there is evidence to suggest that a number of the most notorious Gram-positive pathogenic bacteria, including *Listeria monocytogenes*, *Clostridium botulinum* and *Staphylococcus aureus*, produce related peptides. The distribution of the *L. monocytogenes* cluster is particularly noteworthy in that it is found exclusively among a subset of lineage I strains; i.e., those responsible for the majority of outbreaks of listeriosis. Expression of these genes results in the production of a haemolytic and cytotoxic factor, designated Listeriolysin S, which contributes to virulence of the pathogen as assessed by murine- and human polymorphonuclear neutrophil–based studies. Thus, in the process of establishing the existence of an extended family of SLS-like modified virulence peptides (MVPs), the genetic basis for the enhanced virulence of a proportion of lineage I *L. monocytogenes* may have been revealed.

## Author Summary

*Listeria monocytogenes* is known to produce only one haemolytic factor that plays an important role in its pathogenic cycle—that is, the cholesterol-dependent Listeriolysin O. However, here we identify a second haemolysin, which is present in a subset of strains of lineage I, the evolutionary lineage of *L. monocytogenes* that contributes to the majority of spontaneous and epidemic outbreaks of listeriosis. This second haemolysin is only induced under oxidative stress conditions and contributes to murine virulence and in survival in polymorphonuclear neutrophils. Bioinformatic analysis suggests that the haemolysin Listeriolysin S (LLS) is post-translationally modified and belongs to a family of modified virulence peptides, including the potent virulence factor Streptolysin S (SLS), a peptide cytolysin produced by *Streptococcus pyogenes*, and several as-yet uncharacterised members of the same family in other pathogens. Therefore, en route to establishing that some *L. monocytogenes* produce more than one haemolysin, we have uncovered a virulence factor that is exclusively associated with a subset of the lineage I evolutionary line and that is also the first non-streptococcal representative of a potentially extended family of SLS-like peptides.

## Introduction

**Figure 1:**
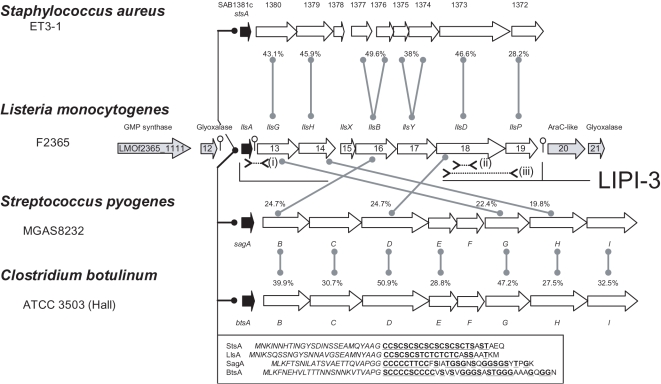
Comparison of MVP-associated operons. Arrangement of the Stapholysin S (*sts*), Listeriolysin S (*lls*), Streptolysin S (*sag*) and Botulysin (*bts*) associated genes in *S. aureus* ET3-1, *L. monocytogenes* F2365, *S. pyogenes* MGAS 8232 and *C. botulinum* ATCC3502 respectively. Homology between the predicted *lls* gene products and their *sag* and *sts* equivalents is indicated by a line and dashed line, respectively, with an associated % identity value. % identity values with respect to the predicted Sag and Bts gene products are also indicated. The amino acid sequence of the predicted unmodified structural peptides, StsA, LlsA, SagA and BtsA is presented (boxed) with italicized letters indicating amino acids likely to constitute the leader region. Residues within the structural propeptide that are potentially modified are underlined. The location of oligonucleotide pairs used to investigate strain variation are indicated (i) llsAint-llsAsoeD, (ii) 1118degf–1118degr and (iii) 1118f–1118r.

Bacteriocins are ribosomally synthesized antibacterial peptides of bacterial origin that have been the focus of much attention since their discovery over 70 years ago. The Class I lantibiotics have been of particular interest, given the unusual post-translational modifications which are required for their activity [1]–[3]. Most modified bacteriocins have been investigated with a view to their potential applications in medicine or food, but there are two exceptional examples of bacteriocin-like modified peptides that are cytotoxic and which contribute to the virulence of bacterial pathogens; i.e. the lantibiotic Cytolysin (*Enterococcus faecalis*) [4] and Streptolysin S (Group A *Streptococcus*; i.e. GAS) [5]. Streptolysin S (SLS) production is particularly significant as it contributes to the cytotoxicity, inflammatory activation and polymorphonuclear neutrophil (PMN) resistance of GAS, thereby playing a role in necrosis and systemic spread [6]. It is also responsible for the associated characteristic β-haemolytic activity. The structure of the SLS peptide has not been elucidated and it was only following identification of the associated genetic determinants that it was realised that SLS is a post-translationally modified bacteriocin-like peptide [7]. More specifically, analysis of the SLS-associated genes (*sag*; *S*treptolysin S *a*ssociated *g*enes) indicates that the structural peptide (encoded by *sagA*) is extraordinarily rich in cysteines (Cys), glycines (Gly) and hydroxylated amino acids (Fig. 1). It has been established that post-translational modifications occur that result in the formation of thiazole and oxazole residues (or heterocycles) [5],[7],[8] in a manner analogous to that associated with the bacteriocin microcin B17 (Mcb) [9]. A number of other proteins which are essential for SLS production [5] are also typical of those associated with bacteriocin biosynthesis or immunity, including most notably SagB, which is homologous to the oxidoreductase McbC required for the post-translational modification of microcin B17 [10]. Indeed, it has been established that SLS biosynthetic enzymes, SagBCD, function in a manner analogous to the microcin B17 modification machinery, McbBCD, as SagBCD can substitute for McbBCD with respect to modification of the microcin B17 structural peptide *in vitro* [8].

It had been thought for some time that SLS-like virulence factors are exclusively associated with streptococci (related peptides are produced by group C and G streptococci and *Streptococcus iniae*) [6]. *In silico* analysis demonstrates that gene clusters potentially encoding Sls-like factors are also present in some of the most notorious Gram positive pathogens, including *Listeria monocytogenes*, *Staphylococcus aureus* and *Clostridium botulinum*. A specific focus on the *L. monocytogenes* cluster has revealed that it encodes an inducible cytotoxic and haemolytic peptide, Listeriolysin S, which plays a role in the survival of the pathogen in PMNs and contributes to its virulence in the mouse model. Notably, the Listeriolysin S-associated cluster is strain variable, being present in a subset of isolates from lineage I, the evolutionary lineage associated with the majority of epidemics associated with this often fatal pathogen.

## Results

### *In silico* screen for novel SLS-like factors

Previous *in-silico* investigations have successfully identified producers of novel post-translationally modified lantibiotics by screening for the presence of associated genes which are likely to be highly conserved; i.e. those encoding proteins that are involved in post-translational modifications [11],[12]. We used a similar approach to identify novel producers of SLS-like peptides, searching for homologues of *sagB*, the gene encoding the McbC-like oxidoreductase associated with heterocycle formation [7]. This revealed that SagB-related proteins are potentially produced by a number of *L. monocytogenes* strains (including the genome-sequenced strains F2365 (NC_002973) and H7858 (NZ_AADR00000000)), by *S. aureus* ET3-1 (a representative of the most abundant bovine mastitis-associated *S. aureus* lineages (NC_007622)), and by a number of group I *Clostridium botulinum* strains (ATCC3502/Hall (NC_009495), ATCC19397 (NC_009697) and Langeland (NC_009699)). In each case the SagB homolog is located in a cluster of additional *sag* homologues. This fact has also been recently noted by Lee et al [8]. Notably, each of these clusters contains a gene capable of encoding a SLS-like peptide (which we designate Listeriolysin S (LLS), Stapholysin S (STS) and Botulysin S (BTS) (Fig. 1)). The putative structural genes (*llsA*, *stsA* and *btsA*) all encode a peptide consisting of a leader region that terminates with the classical ‘double glycine’ bacteriocin-associated leader cleavage motif [13] and a putative pro-peptide with an extreme predominance of cysteine, serine, threonine and, in the case of BTS (and SLS), glycine residues (Fig. 1), all of which are residues involved in heterocycle modification. Notably, the presence of *llsA* in genome-sequenced *L. monocytogenes* has been overlooked during annotation of the relevant genomes. It should be noted that it contrast to the depiction by Lee et al. [8], *llsA* is the first gene within the LLS cluster. Examination of the clusters in greater depth establishes two subgroups consisting of the BTS/SLS and LLS/STS gene clusters (Fig. 1). In the case of the eight *lls* genes, only *llsX* does not have significant identity with its *sts* equivalent, while both *llsB* and *llsY* are represented by two ORFs in the Sts gene cluster (Fig. 1). Our re-sequencing of this region in *S. aureus* ET3-1 has confirmed that these ORFs are not an artefact from genome sequencing of ET3-1. On the basis of genome dissimilarity values and GC content analysis (Table S1), the *lls* and *sts* genes also differ from their *sls* and *bts* counterparts in that they appear to have been acquired by horizontal gene transfer.

We conducted a more in-depth analysis of the LLS gene cluster since it was found in *L. monocytogenes* F2365 and H7858, two strains responsible for epidemic outbreaks of listeriosis (a life-threatening infection of pregnant and immuno-compromised humans [14]). We named the cluster *Listeria* pathogenicity island 3 (LIPI-3), in order to distinguish it from LIPI-1, present in all pathogenic *L. monocytogenes* and containing many of the well established virulence factors associated with pathogenesis [15], and LIPI-2, a pathogenicity island found exclusively in the ruminant pathogen *L. ivanovii* [16]. LIPI-3 is flanked by *Rho*-independent terminators and two related glyoxalase-encoding genes (*lmof2365_1111* and *lmof2365_1121*; 81% identity). The predicted *llsA* promoter (PllsA) does not contain any of the motifs previously associated with virulence gene regulation in other *L. monocytogenes* strains (e.g., a PrfA box or a σ^B^ binding site). In addition to LlsA and LlsB (24.7% identity with SagB), other proteins encoded by LIPI-3 include LlsGH, two components of an ABC transporter (22.4% and 19.8% identical to SagGH, respectively), LlsD (24.7% identity with SagD) and LlsP, which has been annotated as an N-terminal protease (frequently associated with bacteriocin leader cleavage). Given their location within this region, *llsX* and *llsY* are also likely to be relevant (Fig. 1).

### Investigation of the distribution of LIPI-3 in *L. monocytogenes*

**Figure 2:**
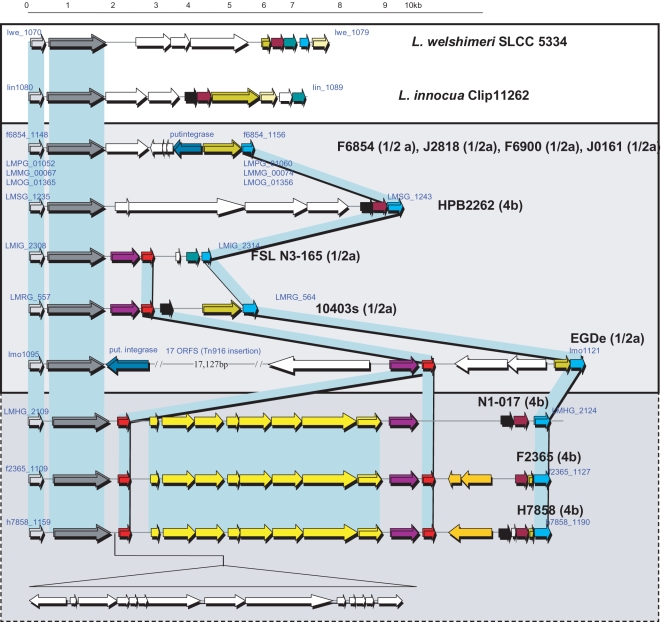
LIPI-3 and corresponding regions in LIPI-3 minus *Listeria*. Comparison of the LIPI-3-containing region (bottom) with the corresponding region of LLS^−^ *L. monocytogenes* (middle), *L. innocua* and *L. welshimeri* [49]. For *L. monocytognes* strains, the strain name and serotype (in brackets) is presented and the designation of the first and last gene in each case is that designated in the corresponding genome sequence. Homologous genes (or in the case of the *llsA-P*, clusters of genes) are presented by matching colours.

**Figure 3:**
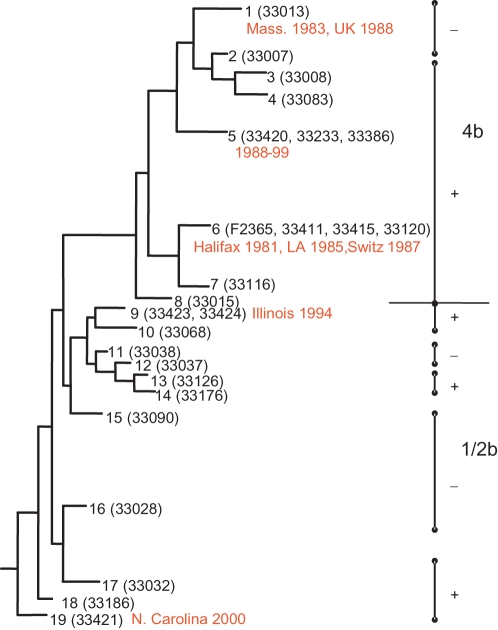
Distribution of LIPI-3 among lineage I *L. monocytogenes*. LIPI-3 status (positive, + or negative, −) mapped onto a maximum parsimony tree inferred from sequence data of lineage I strains (adapted from [24]). The individual sequence types (1–19), representatives tested (strain number in brackets) and associated epidemic outbreaks (in red). Strains of serotype 4b and 1/2b are also distinguished.

*L. monocytogenes* strains can be divided into three evolutionary lineages, I, II and III [17]. Despite the fact that strains of all lineages possess the well established virulence factors associated with the LIPI-1 pathogenicity island [18], lineage I (which consists of strains of serotype 1/2b and the notorious serotype 4b) is most frequently associated with outbreaks and sporadic cases of human listeriosis [19]. However, to date, no additional virulence factor has been specifically associated with lineage I. We carried out additional *in silico* analysis of genome-sequenced *Listeria* which revealed that although the lineage I strains F2365 and H7858 possess LIPI-3, it is absent from the genome-sequenced strains EGDe [20], F6854 [21] (both lineage II), and the non-pathogenic *L. innocua* CLIP 11262 [20] and *L. welshimeri* SLCC5334 [22] (Fig. 2). These investigations also highlighted the variable nature of this region of *L. monocytogenes* genomes. For instance, H7858 possesses an additional 14 ORFs immediately upstream of its LIPI-3 equivalent, while there is a large amount of inserted DNA (>17 kB) in place of LIPI-3 in EGDe (Fig. 2). Analysis of the corresponding regions from recently released sequence data from a number of *L. monocytogenes* strains generated by the Broad Institute emphasises the genetic variability at this locus ([23]; Fig. 2). Such analysis also reveals that homologs of the up- and downstream genes (*lmof2365_1111* and *lmof2365_1120*) are found in some LIPI-3^−^ strains, thereby confirming that the flanking *Rho*-independent terminators mark the outer limits of LIPI-3 (Fig. 2). To better assess the strain variable nature of the island, we examined additional *Listeria* isolates representing a cross-section of all three lineages, as well as other *Listeria* species. These revealed that LIPI-3 is consistently absent from all lineage II and III *L. monocytogenes* and from all other species tested (*L. innocua*, *L. welshimeri*, *L. seeligeri*, *L. ivanovii*, and *L. grayi*) (Table S2). LIPI-3 is also absent from some lineage I strains; of those tested approximately half possess the island (52% positive; 23/44 strains) (Table S3). The LIPI-3^+^ strains corresponded to representatives of 13 of the 19 lineage I sequence types (STs) [24] previously proposed (i.e. STs 3–10, 13–14 and 17–19). In addition to F2365 and H7858, responsible for epidemic outbreaks of listeriosis in California (1985) and the US (multistate; 1998–99), respectively, the LIPI-3^+^ set includes the listeriosis-outbreak strains from Halifax (1981), Lausanne (1987), Illinois (1994) and North Carolina (2000) (Fig. 3; Table S3). In contrast, relatively few lineage I outbreak-associated strains (Massachusetts 1985, UK 1989) lack LIPI-3 (Fig. 3; Table S3). When the LIPI-3 status of the lineage I strains is mapped onto the phylogenetic tree (maximum parsimony tree inferred from combined sequence data) which revealed the presence of the 19 lineage 1 STs [24], it was apparent that although the presence/absence of the island is consistent within individual STs, there are variations within major branches (Fig. 3). This observation is consistent with repeated independent acquisition or loss of LIPI-3. While %GC content and genome dissimilarity values indicate that LIPI-3 has been acquired, this is more likely to have been an event in an ancestral lineage I strain. Therefore the variable nature of LIPI-3 within lineage I is more likely to reflect its loss within specific sequence types (most likely as a consequence of recombination between *lmof2365_1111* and *lmof2365_1121* and looping out of intervening genes). This would represent a recurring theme in *Listeria* evolution as the emergence of non-pathogenic *Listeria* species has been attributed to the loss of LIPI-1 from the primordial species [25].

### LIPI-3 encodes a haemolytic and cytotoxic factor

**Figure 4:**
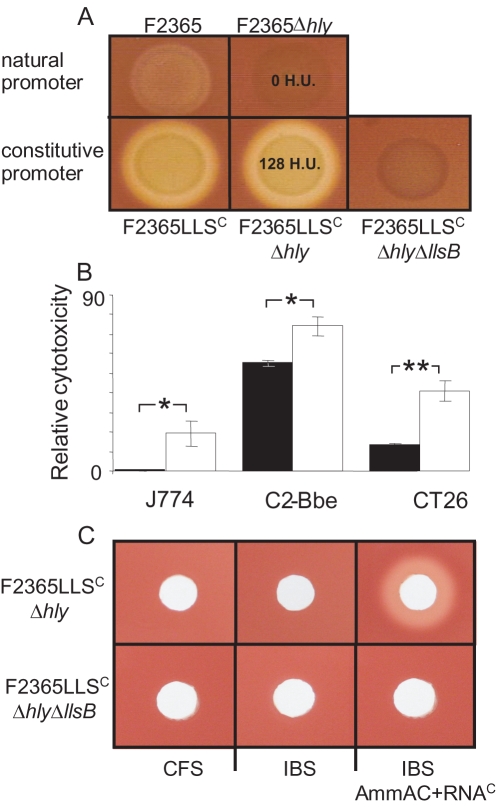
Haemolytic and cytolytic activities of LLS. (A) Haemolysis of Columbia blood agar (5% Sheep's blood) by F2365, F2365Δ*hly* and F2365Δ*hly*Δ*llsB* when the *lls* genes is under the control of the natural PllsA or constitutive P_HELP_ promoters (F2365LLS^C^, F2365Lls^C^Δ*hly* and F2365LLS^C^Δ*hly*Δ*llsB*). In situations where haemolytic activity was quantified, the corresponding haemolytic unit (H.U.) values are presented. (B) Cytotoxicity relative to F2365 (100%) with respect to the J774, C2-Bbe and CT26 cell lines; F2365Δhly-black, F2365LLS^C^Δ*hly*-white. *-significantly different (P<0.05), **-extremely significantly different (P<0.005) different. Error bars represent standard error of the mean. (C) Assessment of the haemolytic activity of cell free supernatant (CFS) of F2365LLS^C^Δ*hly* and F2365LLS^C^Δ*hly*Δ*llsB*, of the cell free supernatant of induction buffer washed F2365LLS^C^Δ*hly* and F2365LLS^C^Δ*hly*Δ*llsB* (IBS), and of IBS from the strains combined with RNA core (RNA^C^; inducer) and ammonium acetate (AmmAc; stabilizer) aliquoted into 4.6 mm wells in Columbia blood agar (5% Sheep's blood). NB. IB, RNA^C^ and AmmAc, both individually and in combination, are non-haemolytic (data not shown).

**Figure 5:**
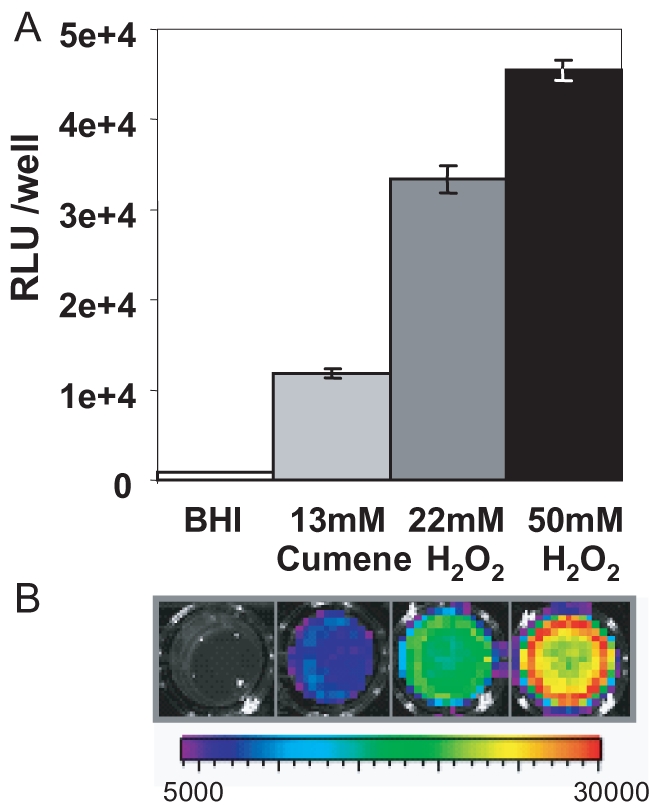
Induction of the PllsA promoter. Induction of PllsA by 13 mM cumene hydroperoxide and 22 and 50 mM hydrogen peroxide. Data are presented as (A) mean relative light units (RLU; photons s^−1^)/well±standard deviations for three replicates, and (B) one representative of three independent experiments is shown. The colour bar indicates bioluminescence signal intensity (in photons s^−1^ cm^−2^).

Since SLS is responsible for the haemolytic activity of GAS, we looked for an association between the presence of LIPI-3 and haemolysis. All *L. monocytogenes* lineages produce the haemolysin Listeriolysin O (a 60 kDa protein encoded by *hly* located within LIPI-1) [15], [26]–[28] which would be expected to mask any haemolytic activity associated with LLS. Therefore, a Δ*hly* mutant of F2365 was created. However, this mutant is non-haemolytic on Columbia blood agar (Fig. 4A). To determine whether the *lls* genes are expressed under these conditions, the Lls promoter (PllsA) was fused to a Lux reporter system in a promoter probe vector (pPLKm2) and integrated as a single copy into the F2365 genome. Real-time analysis with an IVIS100 imager (Xenogen) revealed that under routine laboratory growth conditions expression of Lux is negligible, but that the promoter was strongly induced upon exposure for 10 min to oxidative stress, including cumene hydrogen peroxide and hydrogen peroxide (Fig. 5). The induction is transient, dissipating gradually over a 60 min period. Unfortunately, the requirement for cumene or hydrogen peroxide induction prevents an assessment of any associated haemolytic and/or cytotoxic activities. To overcome this problem, a constitutively strong synthetic Gram positive promoter, P_HELP_ [29], was introduced upstream of the *lls* operon in both F2365 and F2365Δ*hly*. Placing the genes under the control of P_HELP_ significantly enhanced the haemolytic activity of F2365 (F2365LLS^C^) and also resulted in a haemolytic phenotype against the Δ*hly* background (i.e. F2365LLS^C^Δ*hly*) (Fig. 4A). This haemolytic activity was eliminated when *llsB*, predicted to be necessary for the production of mature, active LLS, was mutated in a non-polar fashion (F2365LLS^C^Δ*hly*Δ*llsB*), confirming the link between the LIPI-3 island and the haemolytic phenotype (Fig. 4A). *L. monocytogenes* cells were used in haemolytic assays as it was apparent that LLS, like SLS, is active in a cell-associated form with no haemolytic activity evident from cell-free culture supernatant. Constitutive expression of LLS also resulted in enhanced cytotoxicity as, F2365LLS^C^Δ*hly* was significantly more cytotoxic than F2365Δ*hly* against C2-Bbe (human enterocyte-like), J774 (mouse macrophage) and CT26 (mouse colon carcinoma) cell lines (Fig. 4B).

One of the intriguing features of SLS is that although it is active when cell associated, it is inactive in cell-free situations unless it is activated by an inducer such as RNA core (ribonuclease-resistant fraction of yeast RNA) in a suitable buffer (RNA^C^) [30],[31]. Our analysis of cell-free supernatant (CFS) from F2365LLS^C^Δ*hly* and F2365LLS^C^Δ*hly*Δ*llsB* cultures established that both lacked haemolytic activity (Fig. 4C). However, as with SLS-producers [31], when the LLS-producing cell (F2365LLS^C^Δ*hly*) were washed with an induction buffer (IB), haemolytic activity was apparent if the induction buffer supernatant (IBS) was combined with inducer (RNA^C^) and stabiliser (ammonium acetate buffer; AmmAc). Such haemolytic activity was not induced when F2365LLS^C^Δ*hly*Δ*llsB* cells were similarly treated. These investigations provide further confirmation that LLS is a SLS-like toxin.

### Role of LIPI-3 in the virulence of *L. monocytogenes* F2365

**Figure 6:**
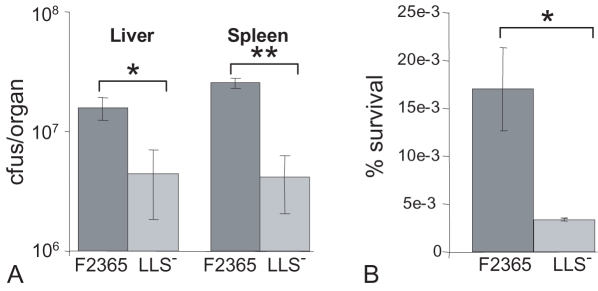
Comparison of the virulence of wildtype F2365 and a LLS^−^ derivative. (A) Levels (cfu–colony forming units) of wild-type (F2365) and mutant (F2365Δ*llsB*; LLS^−^) F2365 in livers and spleens of Balb/C mice 3 days post-intraperitoneal infection. (B) Survival in human PMNs after 2 hr. * - significantly different (P<0.05), ** - extremely significantly different (P<0.005) different. Error bars represent standard error of the mean.

Although it was established that the *lls* genes encode a cytotoxic and haemolytic factor, we wanted to assess whether they contribute to the virulence of *L. monocytogenes* when under the control of their native promoter. Given that the importance of SLS only became apparent upon the analysis of an isogenic mutant [5], we assessed the contribution of LIPI-3 by comparing the virulence of wild-type F2365 and F2365Δ*llsB* (LLS^−^) following intraperitoneal inoculation of Balb/c mice. From these assays it was apparent that the LLS^−^ strain possessed a reduced virulence potential, as evidenced by significantly reduced bacterial levels in the livers and spleens relative to the corresponding wildtype F2365-infected mice (Fig. 6A). We also investigated if LLS contributes to the survival of *L. monocytogenes* in human PMNs. This was prompted by the fact that firstly, SLS contributes to the ability of GAS to withstand neutrophil killing [5]; secondly, of all phagocytes, PMNs produce the greatest concentration of oxidative stress-inducing species (i.e. those which induce PllsA); and thirdly, the initial response to the intraperitoneal infection of mice involves an influx of neutrophils. Notably, PMNs are essential for the resolution of *L. monocytogenes* infections, playing a critical role in reducing the bacterial burden in the liver, spleen and central nervous system [32]. When the intracellular survival of F2365 and LLS^−^ in purified human PMNs was compared after 2 hrs, it was apparent that wild-type F2365 again survived significantly better than the LLS^−^ mutant (Fig. 6B).

## Discussion

This study provides new insights into the pathogenesis of several important Gram positive pathogens. Until recently, modified virulence peptides have been rarely reported and SLS-like cytolysins have been exclusively associated with *Streptococcus* sp.; including group A, C and G streptococci and *S. iniae* (a fish pathogen) (11). It is now apparent that a number of related gene clusters can be identified in other Gram positive pathogens, including *L. monocytogenes*, *S. aureus* and *C. botulinum*. In fact, Lee et al. have recently reported that in addition to these pathogens, more distantly related peptides may be produced by microorganisms spanning six phyla [8]. Discovering the role of these individual peptides, whether it be in conferring antimicrobial (as with microcin B17) or cytolytic activity (as with SLS) or even secondary metabolism/morphogenesis [33] now becomes the next big challenge. With respect to the clusters that most closely resemble that responsible for SLS production, while the functionality of the *S. aureus* and *C. botulinum* gene clusters has yet to be confirmed (although in the latter case *in vitro* production of modified peptide confirms its haemolytic nature [8]), our analysis of LIPI-3 and LLS establishes definitively the existence of an extended family of SLS-like modified virulence peptides (MVPs), which contribute to the general virulence of the producing strains and which specifically aids survival following exposure to PMNs. The association of one such MVP with selected strains of lineage I *L. monocytogenes* may provide an insight into a biologically interesting conundrum associated with this species. Since it was first established that *hly* mutants of *L. monocytogenes* did not haemolyse blood [34], it has been assumed that Listeriolysin O is the sole haemolysin/cytolysin produced by the pathogen. It is now apparent that the absence of LIPI-3 from many strains, including the majority of the most frequently used laboratory strains, coupled with the inducible nature of PllsA, has masked the existence of this second haemolysin/cytolysin.

When one considers their prevalence in animal listeriosis and food contamination isolates, it is apparent that lineage I isolates are overrepresented in human listeriosis cases [19],[35]. However no genetic basis has been suggested which could explain the association between lineage I *L. monocytogenes* and an increased risk of human disease. Efforts to identify an elusive lineage I-specific virulence factor have had to accommodate the fact that such a factor, if it exists, is not a pre-requisite for pathogenesis but would simply confer a higher virulence potential in humans (although perhaps not in animal models of infection). It is tantalising in this regard that genome analyses revealed very few differences between epidemic-associated strains and their non-epidemic associated counterparts. This was first suggested by a genome subtraction study which identified only 39 gene fragments that are present in strain F4565 (4b) but absent from EGD (1/2a) [36]. Our re-examination of these fragments now indicates that three of these 4b specific fragments correspond to regions within LIPI-3. Similarly, following sequencing of the first four *L. monocytogenes* genomes, only 51 serotype 4b-specific genes were identified [21]. Seven of these (plus the previously unannotated *llsA*) are within LIPI-3. However, the homologies between these genes and those involved in SLS biosynthesis have until recently gone unnoticed. This study confirms that these genes encode a haemolytic/cytolytic factor which impacts on virulence potential, and also provide further clarity with respect to their distribution. It is apparent that LIPI-3 is not specific to serotype 4b, but is associated with a subset of lineage I isolates. This distribution pattern is intriguing and strongly implicates this novel haemolysin as a probable candidate for the elusive virulence factor responsible for the association of lineage I strains with spontaneous and epidemic listeriosis outbreaks. We accept that not all cases of listeriosis are associated with LIPI-3^+^ lineage I strains and that a small number of lineage I LIPI-3^−^ strains have also been linked to epidemic listeriosis. This probably reflects the likelihood that any *L. monocytogenes* strain, regardless of the presence or absence of LIPI-3, can cause illness if consumed in sufficient quantities by immunocompromised individuals. Other factors, such as altered expression of other virulence genes [37], may also explain why serotype 4d and 4e strains are not routinely associated with human listeriosis despite, in some cases, possessing LIPI-3. Therefore, while we suggest that LIPI-3 is the best candidate to date to explain the greater association of lineage I with human listeriosis, we anticipate that this hypothesis will no doubt be subjected to rigorous examination by other researchers in the field. It is also anticipated that future studies will reveal the precise mechanism via which LLS contributes to virulence. Given the oxidative-stress inducible nature of PllsA and the contribution of the peptide to neutrophil survival, a role in enabling the survival of any cells still retained in the phagosome upon phago-lysosomal fusion seems the most likely role. Regardless of the outcome of any such assessments, the identification of LIPI-3 and Listeriolysin S is a significant advance in the biology of this important pathogen in that it represents the first pathogenicity island to be associated with this notorious lineage of *L. monocytogenes*.

## Materials and Methods

### Growth conditions

*Listeria* were grown in brain heart infusion (BHI) broth or agar (Oxoid) or Columbia blood agar plates (containing 5% sheep blood; LIP diagnostics, Galway, Ireland) at 37°C unless otherwise stated. Strains used are listed in Tables S2 and S3. *Escherichia coli* EC101 [38] and TOP10 (Invitrogen) were used as intermediate vector hosts. Antibiotics were incorporated as follows: Erythromycin [39], 150 µg/ml *E. coli*, 5 µg/ml *L. monocytogenes*; Chloramphenicol (cm), 10 µg/ml *E. coli* and *L. monocytogenes*; Kanamycin (kan) 50 µg/ml *E. coli*, 25 µg/ml *L. monocytogenes*; Ampicillin (amp) 100 µg/ml *E. coli*. 5-bromo-4-chloro-3-indolyl-b-D-galactopyranoside (X-Gal) was incorporated when required at a concentration of 40 µg/ml.

### Strain variation studies

For strains of unknown lineage, lineage was determined by an allele-specific oligonucleotide PCR multiplex as described previously [24]. The LLS status of strains was determined and confirmed through three distinct PCR reactions involving the primer pairs 1118f–1118r (all primers are listed 5′ to 3′; TCTTACCCTATTATGAAGTATCA, TAACACCATATCCACTTAAATG), 1118degf–1118degr (GATATGTAACTAGCGC TGTATCAACNGGNACNGCT, CCCTCCATACCACCATAAATTAAAKGARTCDATYTG) and llsAint-llsAsoeD (TGCAGCTGGATGTTGCTC, TAGCCCGGGCAGAACTAAAGT). Amplifications were performed in 50 µl volumes with 150 ng/µl concentrations of each primer, 2 mM MgCl_2_, 0.2 mM concentrations of each deoxynucleotide triphosphate, 0.5 U of Taq Polymerase, and 100 ng of genomic DNA. Amplifications consisted of 30 cycles of 1 min at 94°C, 1 min at 56°C, and 1 min at 72°C. Amplification products were resolved on 1.5% (wt/vol) agarose gel.

### Genomic dissimilarity

Compositional bias of dinucleotide frequency analysis using the web-based application deltarho (http://deltarho.amc.uva.nl) [40]. Deltarho calculates the genomic dissimilarity values d_ (the average dinucleotide relative abundance difference) between input sequences and the genome sequence of choice [40]. A high genomic dissimilarity (d_) between an input sequence and the corresponding host genome sequence indicates a heterologous origin of the input sequence.

### Investigation of PllsA expression with a luciferase-based reporter system

The promoter reporter vector pPLK2-lux was generated by amplifying the kanamycin resistance cassette from pTV1-OK [41] with the primer pair KanRF-KanRR (CCCTGCAGGTCGATAAACC, ACGAATTCCTCGTAGGCGC) and the introduction of the *Pst*I-*Eco*RI digested product into similarly-digested pPL2-lux [42]. PllsA was fused with the lux cassette within pPLK2-lux through amplification with PllsAfor-PllsAbluntrev (ATTCGTCGACTTTTGATGCTTAAG, CATTCAAATGCCTCCTTTTTATTT) and cloning into the *Sal*I-*Swa*I sites. The resultant plasmid was isolated from the intermediate host and introduced into F2365. Bioluminescence was investigated by washing overnight cultures and resuspending in an equivalent volume of spent BHI or BHI containing cumene hydrogen peroxide (13 mM) or hydrogen peroxide (22 or 50 mM) and quantified with a Xenogen IVIS 100 imager (Xenogen, Alameda, CA) with a 5 min exposure time.

### Constitutive expression of LLS

To place the *lls* genes under the control of the strong constitutive synthetic promoter P_HELP_, P_HELP_ DNA was amplified with the primer pair PHELPFsoe-PHELPRsoe (GTGGAGTGAAATATAAGTTAGAGG, TCGAGATCTGC AGATGATTGTGATTTAATATTCATGGGTTTCACTCTC) from plasmid pPL2luxPhelp [29] and fused between two DNA fragments amplified from the regions flanking PllsA with the primer pairs PllsAchangeA-PllsAchangeB (ACCTGCAGAAGGGGTTATTGA, CTCTAACTTATATTTCACTCCAC) and PllsAchangeC-PllsAchangeD (ATGAATATTAAATCACAATCATC, TGGAATTCCCAGCTCCATTGTCTC) by SOE (splicing by overlap extension) PCR [43] and cloned into the RepA- shuttle vector pORI280 [44] in the intermediate RepA+ host, *Escherichia coli* EC101 [38]. The resultant pORI280-help vector was introduced into F2365 already containing the RepA+, temperature sensitive helper plasmid pVE6007 [45]. Successfully transformed cells appeared as blue colonies following plating on BHI-Ery-Xgal agar at 30°C. To select for integrants, i.e. cells in which the pORI280-help vector had integrated into the F2365 genome by single crossover homologous recombination, F2365 pVE6007 pORI280-help was grown in BHI-Ery at 30°C, subcultured twice (0.1% inoculum) in BHI-Ery at 42°C and streaked onto BHI-Ery-Xgal at 42°C. The introduction of P_HELP_ upstream of *llsA* in Ery^R^/Cm^S^ colonies was confirmed by PCR.

### Deletion mutagenesis by double crossover homologous recombination

The *hly* gene of F2365 was deleted through the amplification (using the primer pairs hlysoeA-soeB (TGGAATTCCACCTAATGGGAAAGT, GGGTTTCACTCTCCTTCTACA) and hlysoeC-soeD (TGTAGAAGGAGAGTGAAACCCTAGTGTAGATAATCC, AAGCCCGGGACAACTAATC-TGAC), splicing and cloning (into the temperature sensitive shuttle vector pKSV7 [46]) of DNA flanking the gene followed by the introduction of the spliced product through double crossover homologous recombination as described previously [47]. This strategy in combination with the primer pairs, llsBsoeA-soeB (ATTCTAGACAAGGTATAGAAAGG, TTTGCTGTTTCCTTTCTATGTCTG) and llsBsoeC-soeD (CAGACATAGAAAGGAAAC-AGCAAACAAATATTTGTG, TCTCCCGGGAAATAGCTCTTCAC) was also utilized to delete *llsB* from F2365 and F2365Lls^C^Δ*hly*.

### Haemolytic assays

Haemolysis was assessed by spotting 10 µl of overnight cultures onto Columbia blood agar plates and incubating for 24 hr at 37°C. Haemolytic titre assays were carried out as described by Ginsburg et al (Ginsburg et al. JEM 1965), with some minor modification. Washed *Listeria* were concentrated 10 fold i.e. to 2×10^10^ cfu/ml and serially diluted (twofold) in a volume of 0.3 ml of activation buffer (0.005 M maltose, 0.001 M MgSO_4_-7H_2_O, 0.001 M cysteine) and incubated for 10 min at 37°C. ). 0.5 ml of prewarmed sheep's red blood cells (0.2% in activation buffer) was added and the final volume adjusted to 1.0 ml. The tubes were incubated at 37°C for 4 hr, centrifuged and haemolysis was assessed spectrophotometrically with a Softmax Pro spectrophotometer (Molecular Devices) at 420 nm. Haemolytic units were calculated for each strain by taking the inverse of the last dilution to show complete haemolysis.

### Cytotoxicity assays

C2Bbe1 (ATCC CRC-2102), J774 (ATCC TIB-67) and CT26 (ATCC CRL-2638) cells were used for cytotoxicity assays. Cells were maintained in Dulbecco's modified Eagle's medium (DMEM) containing 4.5 g/liter Glutamax (Gibco Laboratories, Grand Island, NY) and 10% fetal bovine serum (Gibco) at 37°C in a 5% CO_2_ atmosphere. C2Bbe1 growth media also contained 1% (vol/vol) nonessential amino acids (Gibco) and 0.01 mg/ml human transferrin (Calbiochem). In advance of assays cells were trypsinized (C2Bbe1 and CT26; Gibco) or scraped (J774), harvested by centrifugation at 400×g for 8 min, and resuspended in media. Cells were seeded onto 24-well flat-bottom tissue culture plates (Sarstedt, Leicester, United Kingdom) at a concentration of 3×10^5^ cells per well. The plates were incubated for approximately 72 h at 37°C in a 5% CO_2_ atmosphere until confluence was reached. Overnight cultures of *L. monocytogenes* were washed and added at a ratio of 100 bacteria∶cell, and the plates were incubated at 37°C in a 5% CO_2_ atmosphere for 6 hrs at which time cell-free supernatant was collected and cytoxicity assayed with the Cytotox 96 Non-Radioactive cytotoxicity assay (Promega, Madison, WI) according to the kit instructions.

### Partial purification and induction of cell-free LLS

Partial purification of LLS was carried out as described by Alouf and Loridan [31]. Briefly, F2365LLS^C^Δ*hly* was grown overnight at 37°C in BHI supplemented with maltose (1% w/v) and sodium bicarbonate (2% w/v; BHI-BM). 50 ml of this culture was inoculated in 2.5 l BHI-BM and grown at 37°C for 5 hr. The culture was centrifuged, the cell free supernatant (CFS) collected for assessment. The cell pellet was washed in 100 mM potassium phosphate buffer pH 7 before resuspension to a final volume of 40 ml in induction buffer (IB; 100 mM KH2PO4, 2 mM MgSO4, 30 mM maltose (pH 7)). A 1 ml volume of this cell suspension was centrifuged and the cell free supernatant (IBS) was also collected for assessment. The remaining 39 ml volume of cell suspension was incubated at 37°C for 5 min before being induced through the addition of 0.5 mg yeast RNA core (RNA^C^; Sigma)/ml of suspension and immediately centrifuged. The supernatant was collected and supplemented with ammonium acetate (AmmAc; 100 mM, pH 7) (IBS+RNA^C^+AmmAc). Haemolytic activity was assessed through the creation of wells (4.6 mm diameter) were in Columbia blood agar plates (5% sheep's blood) and the introduction of 50 µl volumes of the liquid to be assessed. Plates were incubated overnight at 37°C.

### Murine virulence assay

Groups (N = 5) of 15-week-old BALB/c mice were inoculated intraperitoneally with overnight cultures of F2365 or F2365Δ*llsB* resuspended in 0.2 ml of phosphate-buffered saline to a final concentration of 2×10^6^ CFU/ml. Mice were sacrificed 3 days postinfection, and the numbers of *L. monocytogenes* in the livers and spleens of infected animals were determined by plating serial 10-fold dilutions of organ homogenates on BHI agar. All procedures involving the use of animals were approved by the institutional animal care committee and complied with relevant legal guidelines.

### Isolation of human PMNs

Polymorphonuclear neutrophil granulocytes (PMNs) were isolated by a one step procedure based on a method described by English and Andersen [48]. Briefly, 9 ml EDTA anticoagulated peripheral blood obtained from healthy donors was diluted with an equal volume of 0.9% NaCl and carefully overlain on a discontinuous double gradient formed by layering 4 ml of polysucrose/sodium diatrizoate adjusted to a density of 1.077 g/ml (Histopaque 1077) on 4 ml Histopaque 1119 in 15 ml conical centrifuge tubes (Starstedt UK). The tubes were subsequently centrifuged at 700×g for 30 min at room temperature. After centrifugation two distinct leukocyte cell layers (lymphocytes/monocytes and PMNs, respectively) were obtained above the bottom sediment of erythrocytes. The PMN layer was carefully aspirated and washed in DMEM supplemented with 10% fetal calf serum (DMEM-FCS). Centrifugation followed at 300×g for 10 min at room temperature. After two further washes the cells were counted using trypan blue staining solution (Sigma-Aldrich Chemie, Deisenhofen, Germany) and the viability of the cells was confirmed to be above 97%.

### Quantification of intracellular survival of *L. monocytogenes* in human PMNs

Human PMNs were isolated as described above and were adjusted to a final concentration of 1×10^6^ cells/ml in DMEM-FCS. Overnight cultures of *L. monocytogenes* were washed with PBS, and resuspended in DMEM-FCS at a final concentration of 2×10^7^/mL. PMNs and *L. monocytogenes* were combined to yield a final bacteria∶PMN ratio of 20∶1. Incubation was at 37°C in 5% CO_2_. After 30 mins the PMNs were washed, resuspended in fresh medium containing 50 µg/ml of gentamicin (Sigma) to kill extracellular bacteria, and further incubated until T = 2 hrs. Subsequently, the samples were washed with PBS, and cells were lysed by the addition of ice-cold water, serially diluted in PBS, and plated on BHI agar to determine the number of viable intracellular bacteria.

### Statistical analysis

In all cases the differences in mean values were analysed with an independent samples t-test, following testing for conformity of data to assumptions of parametric statistics (Kolmogorov-Smirnov test for normality and Levene's test for equality of variances).

## Supporting Information

Table S1 Genomic dissimilarity and %GC of MVP-encoding islands.
(0.03 MB DOC)

Table S2 LLS status of non-lineage I *Listeria*
(0.11 MB DOC)

Table S3 LLS status of lineage I *L. monocytogenes*
(0.12 MB DOC)
