# Supplementary material for: Listeriolysin S, a Novel Peptide Haemolysin Associated with a Subset of Lineage I Listeria monocytogenes
Source: PLoS Pathog. 2008 Sep 12;4(9):e1000144. doi: 10.1371/journal.ppat.1000144 (PMC2522273; doi:10.1371/journal.ppat.1000144)
Supplement: Table S1 — Genomic dissimilarity and %GC of MVP-encoding islands. (0.03 MB DOC) [file ppat.1000144.s001.doc]

**Table S1.** Genomic dissimilarity and %GC of MVP-encoding islands.

| Island | 1000 x δ* island  (genomic dissimilarity) | 1000 x δ* genome average | % GC Island | % GC Genome |
| --- | --- | --- | --- | --- |
|  |  |  |  |  |
| LLS | 117.8 | 34.3 | 29.9 | 38 |
| STS | 61.3 | 45 | 26.6 | 32.8 |
| SLS | 35.4 | 36.2 | 35 | 38.5 |
| BTS | 33.8 | 33.6 | 26.2 | 28.2 |
