# Supplementary material for: Listeriolysin S, a Novel Peptide Haemolysin Associated with a Subset of Lineage I Listeria monocytogenes
Source: PLoS Pathog. 2008 Sep 12;4(9):e1000144. doi: 10.1371/journal.ppat.1000144 (PMC2522273; doi:10.1371/journal.ppat.1000144)
Supplement: Table S2 — LLS status of non-lineage I Listeria (0.11 MB DOC) [file ppat.1000144.s002.doc]

**Table S2.** LLS status of non-lineage I *Listeria*

| **Lineage^#^** | | | **Strain^†^** | **Equivalent** | **Original source** | **Serotype** | **Lls**^‡^ |
| --- | --- | --- | --- | --- | --- | --- | --- |
| ***L. monocytogenes*** | | | |  |  |  |  |
| II^A^ | 33225a^C^ | | | LMB0455 |  | 3a | -^X^ |
| II^A^ | 33226a^C^ | | | LMB0456 |  | 3c | -^X^ |
| II^B^ | NCTC7973 | | |  | Clinical | 1/2a | -^X^ |
| II^B^ | CD1061 | | |  | Pork sausage | non 4 | -^X^ |
| II^A^ | 33022a^C^ | | | ATCC15313 | Rabbit | 1/2a | -^X^ |
| II^B^ | DPC4605 | | | SLCC2479 | Unknown | 3c | -^X^ |
| II^B^ | CD1198 | | |  | Ground turkey | non 4 | -^X^ |
| II^B^ | CD1038 | | |  | Pork sausage | non 4 | -^X^ |
| II^B^ | CD1059 | | |  | Pork sausage | 1 2 | -^X^ |
| II^B^ | CD241 | | |  | Silage | 3 | -^X^ |
| II^B^ | CD1028 | | |  | Pork sausage | non-4 | -^X^ |
| II^B^ | CD243 | | |  | Silage | 1 /2 | -^X^ |
| II^B^ | CD1742 | | |  | Pork sausage | non-4 | -^X^ |
| II^A^ | EGDe | | |  | Laboratory strain | 1/2a | -^X, Y^ |
| II^A^ | 33234a | | | F6854 | Turkey frankfurter | 1/2a | -^Y^ |
| II^A^ | 10403S | | |  | Laboratory strain | 1/2a | -^Y^ |
| II^A^ | J2818 | | |  | Turkey deli (US 1989) | 1/2a | -^Y^ |
| II^A^ | FSL N3-165 | | |  | Soil | 1/2a | -^Y^ |
| II^A^ | FSL J1-101 | | | 33418, F6900 | Clinical | 1/2a | -^Y^ |
| II^A^ | LO28 | | |  | Laboratory strain | 1/2c | -^Y^ |
| II^A^ | FSL J2-003 | | |  |  | 1/2a | -^Y^ |
| II^A^ | J0161 | | | 33419, FSL R2499 | Turkey deli (US 2000) | 1/2a | -^Y^ |
| II^A^ | FSL F2-515 | | |  |  | 1/2a | -^Y^ |
| III^A^ | 33077a^C^ | | | 98-18140 | Bovine tissue | 4b | -^X^ |
| III^A^ | 33115a^C^ | | | 93-500 | Arabia oryx | 4c | -^X^ |
| III^B^ | CD83 | | |  | Silage | 4 | -^X^ |
| III^A^ | FSL J2-071 | | |  |  | 4c | -^Y^ |
| III^A^ | FSL J1-208 | | |  | Animal clinical | 4a | -^Y^ |
| ***L.innocua*** | |  | |  |  |  |  |
|  | CLIP11262 | | |  |  |  | -^Y^ |
|  | FH2333 | | |  | Lettuce |  | -^X^ |
|  | FH2381 | | |  | Sausage rolls |  | -^X^ |
| ***L. ivanovii*** | |  | |  |  |  |  |
|  | CD293 | | |  |  |  | -^X^ |
|  | CD588 | | |  |  |  | -^X^ |
|  | CD165 | | |  |  |  | -^X^ |
| ***L. grayi*** |  | | |  |  |  |  |
|  | ATCC25403 | | |  | Corn stalks |  | -^X^ |
|  | ATCC25400 | | |  | Corn stalks |  | -^X^ |
|  | FH2289 | | |  | Cooked chicken |  | -^X^ |
| ***L. welshimeri*** | | | |  |  |  |  |
|  | SLCC5334 | | |  |  |  | -^Y^ |
|  | FH2109 | | |  | Chicken |  | -^X^ |
|  | FH1968 | | |  | Caesar salad |  | -^X^ |
|  | ATCC35897 | | |  | Plant material |  | -^X^ |
| ***L. seelegeri*** | | | |  |  |  |  |
|  | CD166 | | |  |  |  | -^X^ |
|  | FH2062 | | |  | Cooked breakfast |  | -^X^ |
|  | CD944 | | |  |  |  | -^X^ |

^#^ Lineage established previously^A^ or during the course of this study^B^

**^†^** Strains acquired from Todd Ward/ARS culture collection^C^

^‡^LLS status (+ or -) determined by laboratory based experiments (X) or bioinformatic analysis (Y; EGDe (NC_003210), CLIP11262 (NC_003212), F6854 (NZ_AADQ00000000), SLCC5334 (NC_008555) or the *Listeria monocytogenes* Sequencing Project, Broad Institute of Harvard and MIT ([http://www.broad.mit.edu](http://www.broad.mit.edu/)) i.e. 10403S (NZ_AARZ00000000), J2818 (NZ_AARQ00000000), FSL N3-165 (NZ_AARM00000000), FSL J1-101 (NZ_AARU00000000), LO28 (NZ_AARY00000000), FSL J2-003 (NZ_AARM00000000), J0161 (NZ_AARW00000000), FSL F2-515 (NZ_AARI00000000), FSL J2-071 (NZ_AARN00000000) and FSL J1-208 (NZ_AARL00000000)).
