# Supplementary material for: Listeriolysin S, a Novel Peptide Haemolysin Associated with a Subset of Lineage I Listeria monocytogenes
Source: PLoS Pathog. 2008 Sep 12;4(9):e1000144. doi: 10.1371/journal.ppat.1000144 (PMC2522273; doi:10.1371/journal.ppat.1000144)
Supplement: Table S3 — LLS status of lineage I L. monocytogenes (0.12 MB DOC) [file ppat.1000144.s003.doc]

***Table S3.*** *LLS status of lineage I L.* monocytogenes

| Lineage^#^ | | Strain^†^ | | Equivalent | | Source | ST* | Serotype | Lls^‡^ |
| --- | --- | --- | --- | --- | --- | --- | --- | --- | --- |
| I^A^ | 33013a^C^ | | ScottA | | Clinical (Mass. outbreak, 1983) | | 1-1a | 4b | -^X^ |
| I^A^ | 33413a^C^ | | Ts45 | | Food (UK outbreak, 1988) | | 1-1b | 4b | -^X^ |
| I^A^ | 33007a^C^ | | RM2218 | | Food | | 1-2a | 4b | -^X^ |
| I^A^ | 33008a^C^ | | RM2387 | | Food | | 1-3a | 4b | +^X^ |
| I^A^ | 33083a^C^ | | F1109 | | Food | | 1-4a | 4b | +^X^ |
| I^A^ | 33420a^D^ | | NI-227, H7738 | | Food (US outbreak, 1998-99) | | 1-5a | 4b | +^X^ |
| I^A^ | 33233a^C^ | | H7858 | | Food (US outbreak, 1998-99) | | 1-5b | 4b | +^X,Y^ |
| I^A^ | 33386a^C^ | | NI-225, H7550 | | Clinical (US outbreak, 1998-99) | | 1-5b | 4b | +^X^ |
| I^A^ | 33104a^C^ | | F2365 | | California 1985 | | 1-6a | 4b | +^X,Y^ |
| I^A^ | 33410a^D^ | | JI-119, TS43, F4565 | | Clinical (L.A. outbreak, 1985) | | 1-6a | 4b | +^X^ |
| I^A^ | 33411a^D^ | | FSL N3-008, TS50, L.4760 | | Food (Halifax outbreak, 1981) | | 1-6b | 4b | +^X^ |
| I^A^ | 33415a^D^ | | FSL N3-022, TS21, L.4486j | | Food (Switzerland outbreak, 1987) | | 1-6c | 4b | +^X^ |
| I^A^ | 33120a | | ATCC19118 | | Animal | | 1-6c | 4e | +^X^ |
| I^A^ | 33116a | | ATCC19117 | | Animal | | 1-7a | 4d | +^X^ |
| I^A^ | 33015a^C^ | | 12375 | |  | | 1-8a | 4b | +^X^ |
| I^A^ | 33424a | | FSL R2-503 | | Clinical (Illinois outbreak, 1994) | | 1-9a | 1/2b | +^Y^ |
| I^A^ | 33423a^C^ | | G6003 | | Food | | 1-9a | 1/2b | +^X^ |
| I^A^ | 33068a^C^ | | 8058 | | Animal | | 1-10a | 1/2b | +^X^ |
| I^A^ | 33038a^C^ | | OB001385 | | Food | | 1-11a | 1/2b | -^X^ |
| I^A^ | 33037a^C^ | | OB001350 | | Food | | 1-12a | 1/2b | -^X^ |
| I^A^ | 33126a^C^ | | 7034 | | Animal | | 1-13a | 1/2b | +^X^ |
| I^A^ | 33176a^C^ | | 20240-954 | | Animal | | 1-14a | 1/2b | +^X^ |
| I^A^ | 33090a^C^ | | 7675 | | Animal | | 1-15a | 1/2b | -^X^ |
| I^A^ | 33028a^C^ | | OB001102 | | Food | | 1-16a | 1/2b | -^X^ |
| I^A^ | 33390a | | FSL J2-064 | | | | 1-16f | 1/2b | -^Y^ |
| I^A^ | 33032a^C^ | | OB001186 | | Food | | 1-17a | 1/2b | +^X^ |
| I^A^ | 33186a^C^ | | 20674-01 | | Animal | | 1-18a | 1/2b | +^X^ |
| I^A^ | 33421^C^ | | J0144 | | Food (Nrth Car. outbreak, 2000) | | 1-19a | 4b** | +^X^ |
| I^A^ | CD1121 | |  | | Ground beef | | nd | 4b | -^X^ |
| I^A^ | F5817 | |  | | Clinical (Mass. outbreak, 1983) | | Nd | non 4 | -^X^ |
| I^B^ | CD2088 | |  | | Pork sausage | | Nd | 4b | -^X^ |
| I^A^ | FSL J1-220 | | | | Clinical (Mass. outbreak, 1983) | | Nd | 1/2b | +^X^ |
| I^B^ | DPC4608 | | SLCC1694 | | Unknown | | Nd | non-4 | -^X^ |
| I^B^ | CD1032 | |  | | Pork sausage | | Nd | 4a | -^X^ |
| I^B^ | CD147 | |  | | Dairy enrichment | | Nd | non 4 | +^X^ |
| I^B^ | CD1066 | |  | | Pork sausage | | Nd | non-4 | -^X^ |
| I^B^ | CD749 | |  | | Ground beef | | Nd | non-4 | -^X^ |
| I^B^ | CD748 | |  | | Ground beef | | Nd | 4 | -^X^ |
| I^B^ | CD246 | |  | | Silage | | Nd | 4 | -^X^ |
| I^B^ | CD878 | |  | | Clinical | | Nd | 1 2 | +^X^ |
| I^B^ | CD1078 | |  | | Chicken | | Nd | 1/2b | - ^X^ |
| I^A^ | FSL J1-175 | | | | Water | | Nd | 1/2b | -^Y^ |
| I^A^ | FSL J1-194 | | | | Sporadic clinical | | Nd | 4b | +^Y^ |
| I^A^ | FSL N1-017 | | | | Trout in brine | | Nd | 4b | -^Y^ |
| I^A^ | HPB2262 | |  | | Febrile cases (Italy) | | Nd | 4b | -^Y^ |

# Lineage established previously^A^ or during the course of this study^B^

**^†^** Strains acquired from Todd Ward/ARS (International Life Sciences Institute)^C^ or Martin Wiedman/ILSI (International Life Sciences Institute)^D^ culture collections.

* ST (Sequence Type) as determined previously (1).

^‡^LLS status (+ or -) determined by laboratory based experiments (X) or bioinformatic analysis (Y; F2365 (NC_002973), H7858 (NZ_AADR00000000) or the *Listeria monocytogenes* Sequencing Project, Broad Institute of Harvard and MIT (http://www.broad.mit.edu) i.e. FSL J2-064 (NZ_AARO00000000), FSLJ2-175 (NZ_AARK00000000), FSL R2-503 (NZ_AARR00000000), FSL J1-194 (NZ_AARJ00000000), HPB 2262 (NZ_AATL00000000) and FSL N1-017 (NZ_AARP00000000)).

** Serotype 4b but part of the 1/2b complex (1)

1. Ducey T F, Page B, Usgaard T, Borucki M K, Pupedis K, Ward T J (2007) A single-nucleotide-polymorphism-based multilocus genotyping assay for subtyping lineage I isolates of *Listeria monocytogenes*. *Appl Environ Microbiol* 73: 133-147.
